# Kinetic and thermodynamic control of butyrate conversion in non-defined methanogenic communities

**DOI:** 10.1007/s00253-015-6971-9

**Published:** 2015-09-25

**Authors:** H. Junicke, M. C. M. van Loosdrecht, R. Kleerebezem

**Affiliations:** Department of Biotechnology, Delft University of Technology, Julianalaan 67, 2628, BC, Delft, The Netherlands

**Keywords:** Syntrophic butyrate conversion, Interspecies electron transfer, Hydrogen inhibition, Thermodynamic control, Kinetic control, Reduced product formation

## Abstract

Many anaerobic conversions proceed close to thermodynamic equilibrium and the microbial groups involved need to share their low energy budget to survive at the thermodynamic boundary of life. This study aimed to investigate the kinetic and thermodynamic control mechanisms of the electron transfer during syntrophic butyrate conversion in non-defined methanogenic communities. Despite the rather low energy content of butyrate, results demonstrate unequal energy sharing between the butyrate-utilizing species (17 %), the hydrogenotrophic methanogens (9–10 %), and the acetoclastic methanogens (73–74 %). As a key finding, the energy disproportion resulted in different growth strategies of the syntrophic partners. Compared to the butyrate-utilizing partner, the hydrogenotrophic methanogens compensated their lower biomass yield per mole of electrons transferred with a 2-fold higher biomass-specific electron transfer rate. Apart from these thermodynamic control mechanisms, experiments revealed a ten times lower hydrogen inhibition constant on butyrate conversion than proposed by the Anaerobic Digestion Model No. 1, suggesting a much stronger inhibitory effect of hydrogen on anaerobic butyrate conversion. At hydrogen partial pressures exceeding 40 Pa and at bicarbonate limited conditions, a shift from methanogenesis to reduced product formation was observed which indicates an important role of the hydrogen partial pressure in redirecting electron fluxes towards reduced products such as butanol. The findings of this study demonstrate that a careful consideration of thermodynamics and kinetics is required to advance our current understanding of flux regulation in energy-limited syntrophic ecosystems.

**Electronic supplementary material:**

The online version of this article (doi:10.1007/s00253-015-6971-9) contains supplementary material, which is available to authorized users.

## Introduction

Anaerobic conversion of fatty acids, such as butyrate, involves a close interaction of different microbial groups. Butyrate-oxidizing bacteria convert 1 mol of butyrate to 2 mol of acetate and hydrogen. This reaction is energetically feasible only by product removal mediated by acetoclastic and hydrogenotrophic methanogens. Such mutually dependent microbial consortia are referred to as syntrophic communities (Kleerebezem and Stams 2000; Schink 1997; Stams 1994). Both, the hydrogen and acetate transferred between these syntrophic partners, serve as electron carriers with carbon dioxide and methane being the final products.

The control of the electron transfer in methanogenic ecosystems is not yet fully understood. Only few studies have focused on flux regulation in syntrophic communities that are active at the thermodynamic boundary of life and share the little amount of energy available. Two methanogenic coculture studies investigated the bioenergetics of either butyrate or ethanol degradation; however, they have been performed in batch mode (Dwyer et al. 1988; Seitz et al. 1988). A lack of adaptation of the syntrophic partner organisms to batch reactor conditions may cause lag periods, reduced activity, or the uncoupling of syntrophic growth which leads to unreliable starting conditions. These bottlenecks can be overcome by continuous reactor operation. Seitz et al. (1990a, b) give examples for continuous syntrophic coculture studies on ethanol. Analyzing the thermodynamic system state during syntrophic ethanol conversion, Seitz et al. (1990b) found an unequal distribution of the total Gibbs energy change among the hydrogen-producing acetogen (23 %) and the hydrogenotrophic methanogen (77 %). Smith and McCarty (1989a, b) performed ethanol perturbations of propionate and ethanol-fed enrichments to study the kinetic and thermodynamic control of reduced product formation such as propanol and long-chained fatty acids. However, energy sharing between the different microbial groups was not further investigated.

**Fig. 1:**
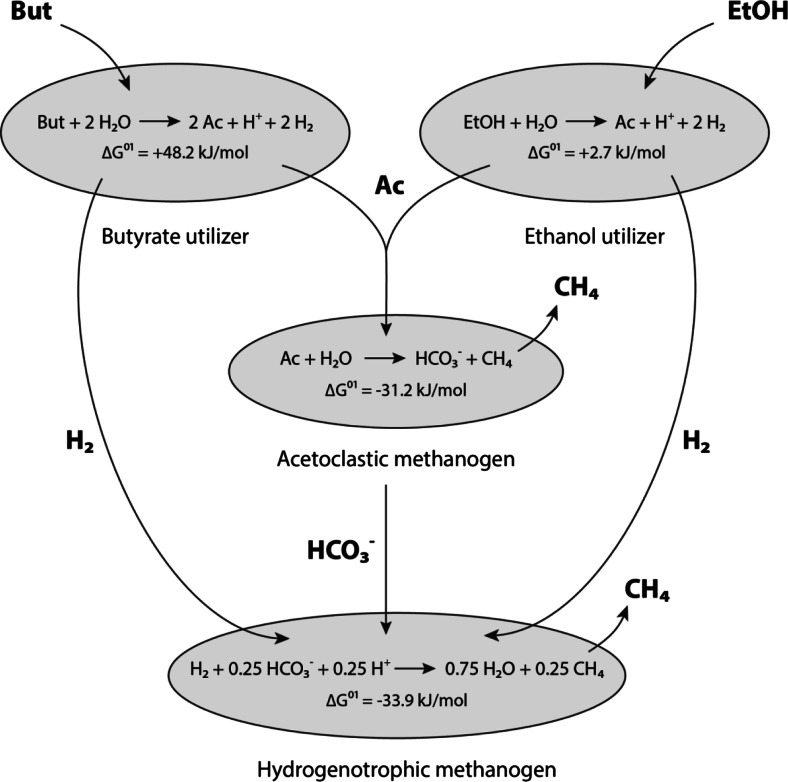
Syntrophic interactions during anaerobic conversion of butyrate and ethanol in non-defined methanogenic associations. *ΔG* ^*01*^ Gibbs energy change under standard conditions and pH 7.0 (kJ/mol donor), *But* butyrate, *EtOH* ethanol, *Ac* acetate

This study aims to elucidate the kinetic and thermodynamic control mechanisms of the electron transfer during syntrophic butyrate conversion in non-defined methanogenic communities. For this purpose, butyrate and ethanol-fed continuously stirred tank reactors (CSTRs) were perturbed with increased ethanol concentrations either at bicarbonate-limiting or non-limiting conditions. The relation of the functional groups participating in syntrophic butyrate and ethanol conversion are shown in Fig. 1

## Material and methods

### Experimental setup and conditions

A double-jacket CSTR (2-L working volume, Applikon, Schiedam, The Netherlands) was inoculated with ground (1 min, 13,500 rpm, Ultra-Turrax T25, IKA-Labortechnik) and sieved (pore size 0.125 mm, Retsch GmbH) anaerobic sludge (Corbion Purac B.V., Gorinchem, The Netherlands) at a final concentration of 40.650 mmol L^−1^. The reactor was operated through five retention times until steady-state conditions were reached, followed by perturbation experiments. The medium composition was according to Junicke et al. (2015a), except for the ammonium concentration (5 mM) and the concentration of the energy sources (20 mM sodium butyrate, 20 mM ethanol). For the perturbation experiments, referred to as C1 and C2, the same influent concentration of butyrate was supplied but the influent concentration of ethanol was increased 5-fold (100 mM). Constant dilution rates of 0.0040 h^−1^ (experiment C1) and 0.0032 h^−1^ (experiment C2) were used. Furthermore, the perturbation experiments differed by the type of base supplied for pH control (0.5 M of NaOH in experiment C1 and 0.5 M of NaHCO3 in experiment C2), while HCl (0.5 M) served as acid in both experiments.

Anaerobic conditions in the reactor liquid were maintained by continuous sparging with dinitrogen gas (0.050 L_N_ min^−1^). The temperature in the reactor liquid was 37 °C and the pH was maintained at 7.2 ± 0.2. Off-gas cooling at 4 °C prevented evaporation and loss of liquid compounds. Temperature, pH, stirring speed (400 rpm), and the inflow of the dinitrogen gas were controlled by a Biostat B plus system (Sartorius Systems, Bohemia, NY). The MFCS/win software program served for data acquisition.

### Analytical measurements

Liquid samples were analyzed by high-performance liquid chromatography (HPLC with Animex HPX-87H column, Bio-Rad; UV detector 2489 and RI detector 2414, Waters, Milford, MA, USA) after filtration through a 0.45-mm pore size filter (Millex-HV filter, Durapore PVDF membrane). The Focus gas chromatograph (Thermo Electron Corporation), equipped with a flame ionization detector and a Hewlett Packard HP INNOWAX 30-m column, was used for the measurement of alcohols. Pentanol served as an internal standard. The Agilent 490 micro gas chromatograph was used for continuous online off-gas analysis, featuring a thermal conductivity detector, a CP-Molsieve channel for H_2_ and a PPQ channel for CH_4_ and CO_2_ measurements. Argon 5.0 served as carrier gas. The total gas outflow rate was obtained by correcting the nitrogen inflow rate for the mole fractions of all the gases produced. The net production rates of each gas (mmol h^−1^) were calculated as the product of the total gas outflow rate and the mole fraction of the respective gas. Cumulative gas amounts were obtained after integrating the net production rates of each gas.

### Molecular techniques

In order to identify the reactions catalyzed by the enriched microbial species in both experiments, the microbial composition was analyzed using denaturing gradient gel electrophoresis (DGGE). Prior to the perturbation with increased ethanol concentrations, samples were taken from the reactor liquid and centrifuged (Junicke et al. 2014). The resulting pellet was stored at −20 °C prior to further analysis. DNA was extracted according to Pronk et al. (2015) and amplified by PCR. For the amplification of the archaeal 16S rDNA gene, the same universal primer set and PCR program were used as previously reported by Pronk et al. (2015). To amplify the bacterial 16S rDNA gene, the same universal primer set and PCR program were used as reported by Bassin et al. (2011), except for the annealing temperature of 55 °C and the elongation phase (72 °C for 30 s).

DGGE analysis of archaeal PCR products was performed according to Pronk et al. (2015), while the DGGE analysis of bacterial PCR products was conducted according to Bassin et al. (2011), except for the use of a different nucleic acid staining solution (SYBR® Gold from Molecular Probes, Eugene, OR). Re-amplification of excised fragments was performed using archaeal and bacterial primer sets and conditions, respectively. Sequencing was conducted by BaseClear B.V. (The Netherlands) and the sequences obtained have been submitted to GenBank under the following accession numbers: KR349066–KR349094.

### Carbon and electron balances

Carbon and electron balances were evaluated at each liquid sampling point to ensure the identification of all compounds and thus an accurate measurement. In a continuous system, the expected total amount of carbon (electrons) at any time equals the measured initial amount in the reactor, plus the amount of carbon (electrons) entering the reactor, minus the amount leaving the reactor, until that time. To express the amount of carbon in carbon moles (C-mol), the amount of all measured compounds was multiplied by the number of carbon atoms per compound. Accordingly, the amount of all measured compounds was multiplied by their degree of reduction (e-mol/mol-compound) to express the amount of electrons in electron moles (e-mol) (Heijnen and Kleerebezem 2010). To obtain the carbon (electron) gap in percent at any time, the difference between the measured and expected total amount of carbon (electrons) was divided by the expected amount of carbon (electrons).

### Descriptive model

Mass balances were used to determine the net conversion rates of each compound, ${R}_i^{net}$ (mmol h^−1^), according to

$$ \frac{R_i^{net}}{V_{\mathrm{R}}}=\frac{\mathrm{d}{C}_i}{dt}-D\left({C}_{\mathrm{in}}-{C}_i\right) $$

where *C*_*i*_ (mmol L^−1^) is the measured concentration of compound *i* in the reactor, *V*_R_ (L) the constant reactor volume, and *D* (h^−1^) the dilution rate. Using estimated stoichiometric yields, the *R*^net^ vector was decomposed into individual reaction rates, *R*_*j*_ (mmol h^−1^). A linear least-squares minimization was performed to obtain those *R*_*j*_ that govern the optimum solution for the defined set of equations

$$ \sum_j{M}_{ij}\bullet {R}_j={R}_i^{net} $$

where *M*_*ij*_ is the stoichiometric matrix element representing compound *i* and reaction *j*. The optimized rate vector, *R*, forms the basis for subsequent model calculations: Compound concentrations in the reactor liquid were derived by step-wise integration of the governing rate equations, *M*∙*R*, and gas production was included by considering gas-liquid mass transfer.

#### Stoichiometric yields

Stoichiometric yields were estimated according to the Gibbs energy dissipation method proposed by Kleerebezem and Van Loosdrecht (2010). Butyrate, ethanol, and hydrogen were assumed as energy source for the butyrate-utilizing species, ethanol-utilizing species, and hydrogenotrophic methanogens, respectively. Acetate was assumed as carbon source for growth in all metabolic reactions considered in this study.

#### Gas-liquid mass transfer

By applying standard mass transfer theory (Cussler 1997), the mass transfer rate (*MTR*, mmol L^−1^ h^−1^) of methane, hydrogen and carbon dioxide was determined as

$$ MTR={k}_{\mathrm{L}}a\ \left(c-{c}^{*}\right), $$

where *k*_L_*a* is the mass transfer coefficient (h^−1^), *c* the gas concentration in the liquid phase, and c^*^ the gas solubility in the liquid. The solubility of carbon dioxide, methane, or hydrogen was derived from the partial pressure of each gas in the reactor headspace and the respective Henry coefficient at 37 °C. The *k*_L_*a* was determined from *k*_L_*a* measurements with oxygen at 37 °C and 400 rpm after correction for the different diffusion coefficients (Cussler 1997; de Kok et al. 2013). When converting measured gas concentrations in the reactor headspace to dissolved gas concentrations in the reactor liquid, oversaturation was considered. The dissolved gas concentration was obtained by multiplying c^*^ with the saturation factor (*c*/c^*^). The saturation factor can be calculated when assuming *pseudo* steady-state conditions, at which the *MTR* is equal to the measured net production rate of the respective gas.

#### Biomass-specific conversion rates

The biomass-specific conversion rate of reaction *j*, *q*_*j*_ (mol (mol-*X*_*j*_)^−1^ h^−1^), was obtained by dividing the optimized *R*_*j*_ by the model-derived biomass amount of the species catalyzing the respective reaction, *N*_*X*_ (mol-*X*_*j*_). The model-derived biomass amount of each species equals the initial biomass amount of that species plus the model-predicted biomass increase. Initial biomass amounts of each species were obtained from the measured total biomass amount at the start of the experiment and the theoretical biomass distribution according to the yield estimation. The total biomass amount was obtained from the measurement of volatile suspended solids and is described elsewhere (Junicke et al. 2014).

#### Determination of hydrogen inhibition constants

The non-competitive inhibition constants of hydrogen on butyrate and ethanol conversion (*K*_*i*H2,C4ox_ and *K*_*i*H2,EtOHox_) were calculated according to

$$ {q}_{\mathrm{S}}={q}_{\mathrm{S}, \max }\ \frac{K_{\mathrm{i}}}{K_{\mathrm{i}}+{c}_{\mathrm{i}}} $$

where *q*_S_ denotes the biomass-specific substrate conversion rate, *q*_S,max_ the maximum biomass-specific substrate conversion rate, and *c*_*i*_ the inhibitor concentration. The *K*_*i*H2,EtOHox_ was obtained by fitting the equation to experiment C1 in the range of 11–72 h using the Levenberg–Marquardt algorithm. *K*_*i*H2,C4ox_ was determined from experiment C2 by solving the equation in the region of constant hydrogen partial pressure, i.e., during steady state and in the range of 62–65 h.

#### Thermodynamic calculations

The actual Gibbs energy change (Δ*G*^1^) of all reactions considered in this study was calculated using

$$ \varDelta {G}^1=\varDelta {G}^{01}+RT\sum {Y}_i \ln {c}_i, $$

where Δ*G*^01^ is the Gibbs energy change at 310.15 K and pH 7.0, *Y*_*i*_ the stoichiometric coefficient of compound *i*, *R* the gas constant (8.314 J K^−1^ mol^−1^), *T* the temperature in Kelvin, and *c*_*i*_ the concentration of compound *i*. The values for the standard Gibbs energies of formation were taken from Hanselmann (1991). The Gibbs-Helmholtz equation was used for temperature correction of Δ*G*^01^ (Kleerebezem and Van Loosdrecht 2010).

## Results

To investigate the kinetic and thermodynamic control mechanisms of anaerobic butyrate conversion, two perturbation experiments, C1 and C2, were performed using the enrichment on butyrate and ethanol from continuously stirred tank reactors. During steady-steady operation, the influent concentration of butyrate and ethanol was set to 20 mM each. The perturbation experiment was initiated by a 5-fold increase of the ethanol concentration in the influent. Both perturbation experiments differ by the dilution rate, either 0.0040 h^−1^ (C1) or 0.0032 h^−1^ (C2), and the base used for pH control, either sodium hydroxide (C1) or bicarbonate (C2). The latter was used to prevent bicarbonate limitation of hydrogenotrophic methanogenesis as observed during experiment C1.

In both experiments, the carbon and electron balances showed a gap of less than 5 % on average. This implies that all compounds were identified and measured accurately. The metabolic reactions involved in syntrophic butyrate and ethanol conversion are shown in Table 1 and were estimated by means of the Gibbs energy dissipation method proposed by (Kleerebezem and Van Loosdrecht 2010).

**Table 1:** Metabolic reactions involved in the syntrophic conversion of butyrate and ethanol as derived from the Gibbs energy dissipation method according to Kleerebezem and Van Loosdrecht (2010)

No.	Reaction
1	$\mathrm{But}+1.971\ {\mathrm{H}}_2\mathrm{O}+0.012{\ NH}_4^{+}\to 1.971\ Ac+0.982\ {\mathrm{H}}^{+}+1.994\ {\mathrm{H}}_2+0.058\ {\mathrm{X}}_{\mathrm{C}4\mathrm{ox}}$
2	$\mathrm{EtOH}+0.944\ {\mathrm{H}}_2\mathrm{O}+0.022{\ NH}_4^{+}\to 0.944\ Ac+0.966\ {\mathrm{H}}^{+}+1.989\ {\mathrm{H}}_2+0.112\ {\mathrm{X}}_{\mathrm{EtOHox}}$
3	$Ac+0.024\ {\mathrm{H}}^{+}+0.919\ {\mathrm{H}}_2\mathrm{O}+0.018{\ NH}_4^{+}\to {0.958\ HCO}_3^{-}+0.954\ {CH}_4 + 0.088\ {\mathrm{X}}_{Acm}$
4	${\mathrm{H}}_2+{0.250\ HCO}_3^{-}+0.254\ {\mathrm{H}}^{+}+0.007\ Ac+0.003{\ NH}_4^{+}\to 0.250\ {CH}_4 + 0.756\ {\mathrm{H}}_2\mathrm{O}+0.015\ {\mathrm{X}}_{Hym}$

The calculation of stoichiometric yields was based on the following conditions: pH 7.0, 298 K, metabolite concentrations from steady-state operation, except for [But] = 2 mM and [EtOH] = 2 mM. Biomass composition according to CH_1.8_O_0.5_N_0.2_ for all species

*But* butyrate, *EtOH* ethanol, *ButOH* butanol, *Ac* acetate, *X*_*C4ox*_ biomass of butyrate-utilizing species catalyzing reaction 1, *X*_*EtOHox*_ biomass of ethanol-utilizing species catalyzing reaction 2, *X*_*Acm*_ biomass of acetoclastic methanogens catalyzing reaction 3, *X*_*Hym*_ biomass of hydrogenotrophic methanogens catalyzing reaction 4

To confirm the occurrence of reactions assumed in the model, bacterial and archaeal 16S rDNA genes were analyzed by DGGE (see Online Resource Fig. S1). A similar microbial composition was found in both experiments: The butyrate-utilizing bacterium enriched in this study (bands 4 and 8) showed 98 % similarity with *Syntrophomonas cellicola* strain 19J-3 (Wu et al. 2006). Several *Methanobacterium* species such as *Methanobacterium flexile* strain GH (100 % gene similarity) and *Methanobacterium subterraneum* strain A8p (97 % gene similarity) were identified as hydrogenotrophic methanogens by DGGE analysis (bands 18, 20, and 29) in this study (Kotelnikova et al. 1998; Zhu et al. 2011). The enriched ethanol-utilizing species (bands 3 and 6) showed 93 % gene similarity with *Pelobacter acetylenicus* strain WoAcy1 (Schink 1985). The identified organisms are known to catalyze the catabolic reactions proposed in Fig. 1, except for acetoclastic methanogenesis. Neither acetoclastic methanogens nor acetate-oxidizing syntrophs were detected using DGGE. Nevertheless, acetoclastic methanogenesis was assumed as the acetate consuming reaction in the model, since phase-contrast micrographs indicated the presence of *Methanosaeta*-like species (see Online Resource Fig. S2).

### Experiment C1

**Fig. 2:**
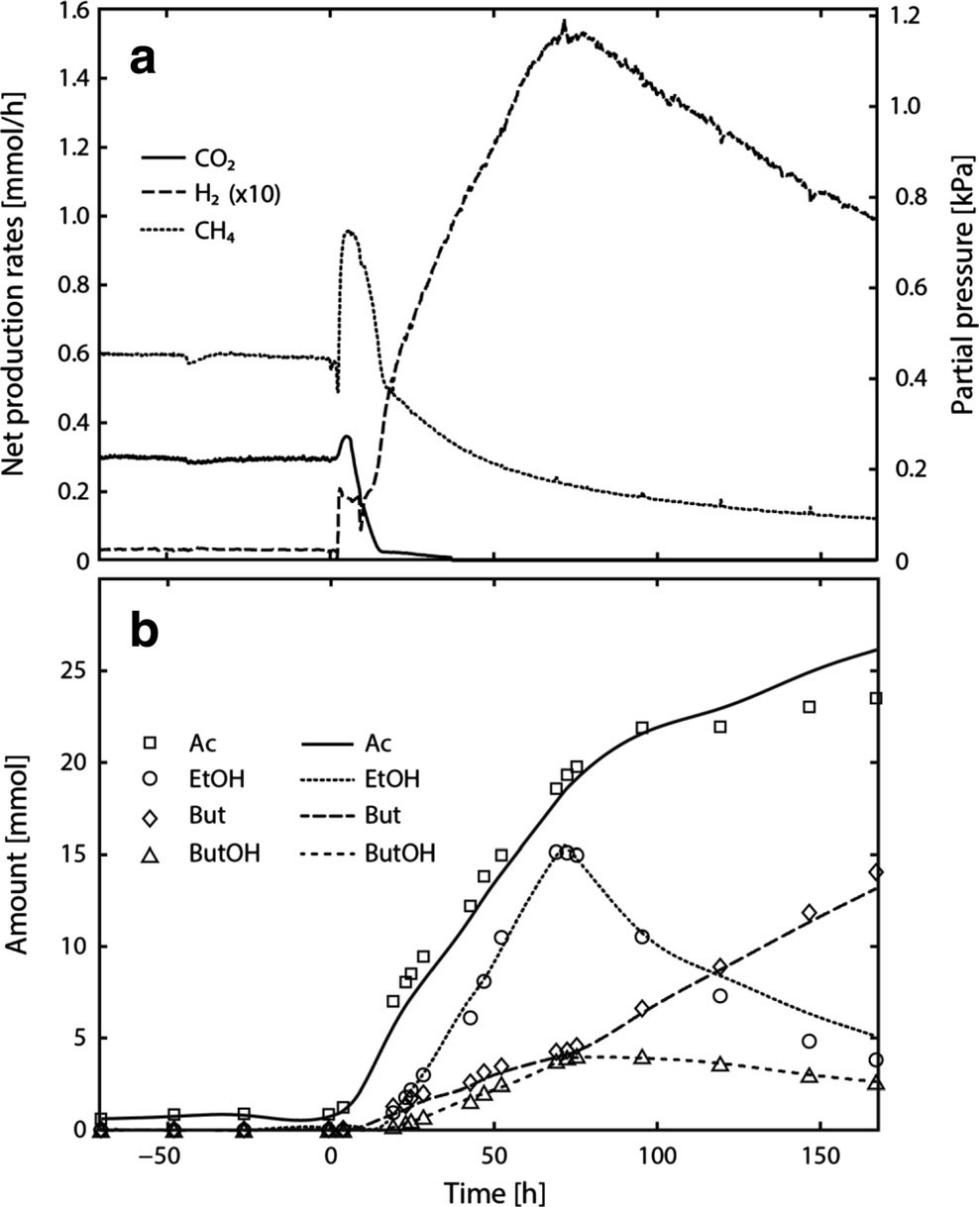
Measured gas production rates (**a**) and amounts in the reactor liquid (**b**) in course of experiment C1. The reactor was operated at a dilution rate of 0.0040 h^−1^ on 20 mM butyrate and 20 mM ethanol using a methanogenic enrichment. Between 0 and 70 , the influent concentration of ethanol was 100 mM, marking the perturbation experiment. In (**a**), the actual hydrogen partial pressure and hydrogen net production rate are obtained by dividing the displayed value by a factor of 10. In (**b**), measured amounts are indicated by *symbols* and model-derived amounts are represented by *lines*

Figure 2a shows the measured net production rates of methane, hydrogen, and carbon dioxide in course of experiment C1, and Fig. 2b shows the model-derived and measured amounts of butyrate, ethanol, acetate, and butanol. Model-derived individual biomass amounts in course of experiment C1 can be found in the Online Resource Fig. S3. At steady state, syntrophic butyrate and ethanol conversion occurred which was reflected in a CH_4_/CO_2_ ratio of about two. The hydrogen partial pressure amounted to 2.4 ± 0.1 Pa. Following perturbation (0 h), the methane and carbon dioxide partial pressures peaked at 720 and 270 Pa. The carbon dioxide partial pressure decreased to zero, 37 h after the perturbation. Contrary to that, the hydrogen partial pressure increased throughout the perturbation experiment, peaked at 120 Pa (70 h) and decreased again after restoring steady-state concentrations of ethanol in the influent.

Interestingly, butanol (2 mM) was formed during the perturbation experiment (Fig. 2b), concomitant with the accumulation of ethanol (7 mM), butyrate (2 mM), and acetate (10 mM). Acetate accumulated shortly after the perturbation event, whereas significant accumulation of the remaining compounds occurred with a delay of 20 h. Ethanol and butanol concentration decreased towards the end of the perturbation experiment. Butyrate, however, continued to accumulate in the reactor and acetate accumulated at a lower rate.

**Fig. 3:**
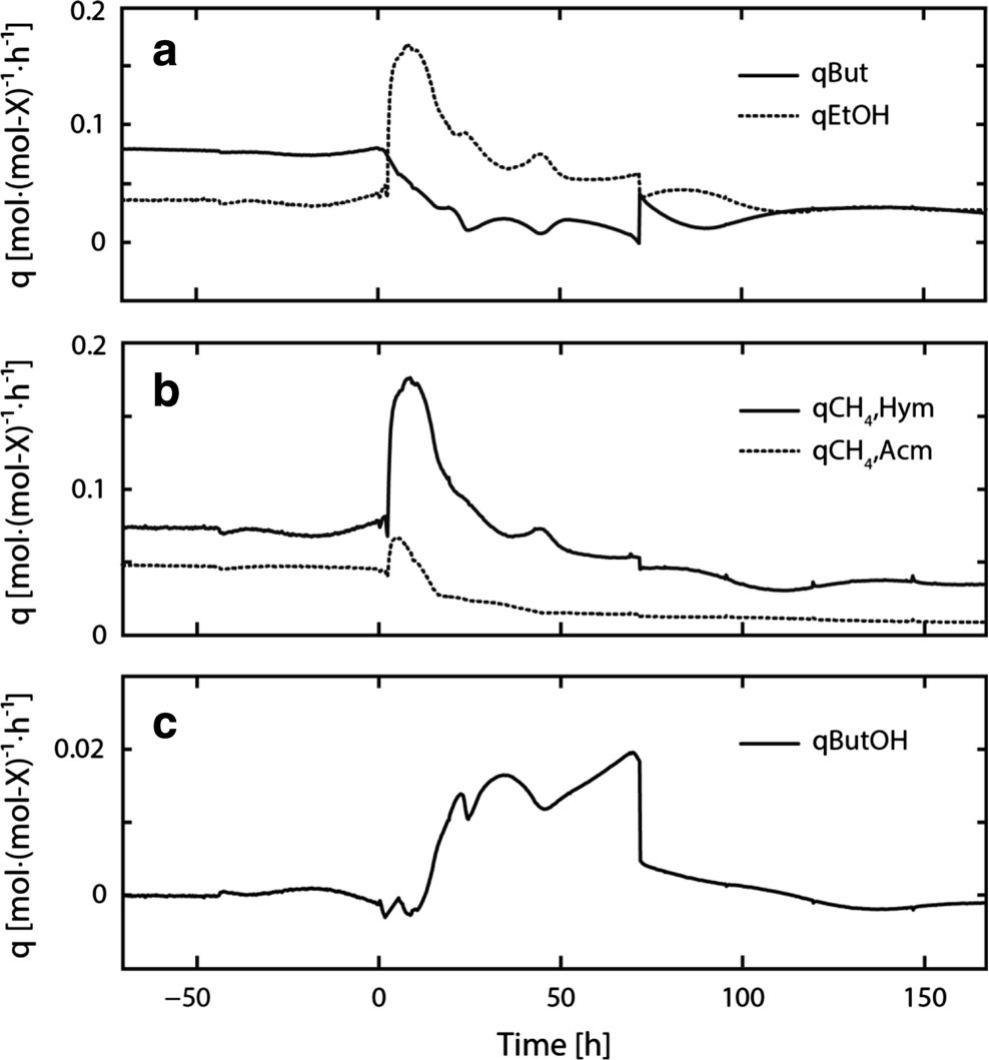
The biomass-specific butyrate consumption rate (*q*_But_) and biomass-specific ethanol consumption rate (*q*_EtOH_) in course of experiment C1 are shown in (**a**). The biomass-specific methane production rate of hydrogenotrophic methanogens (*q*_CH4,Hym_) and biomass-specific methane production rate of acetoclastic methanogens (*q*_CH4,Acm_) are shown in (**b**). The biomass-specific butanol production rate (*q*_ButOH_) is shown in (**c**)

Figure 3 shows the biomass-specific conversion rates in course of experiment C1. Following perturbation, the biomass-specific butyrate consumption rate (*q*_But_) decreased to about zero (24 h) and remained low even after restoring the initial ethanol concentrations in the influent (Fig. 3a). Contrary to that, the biomass-specific ethanol consumption rate (*q*_EtOH_), the biomass-specific methane production rate of hydrogenotrophic methanogenesis (*q*_CH4,Hym_), and the biomass-specific methane production rate of acetoclastic methanogenesis (*q*_CH4,Acm_) increased, reaching their respective maxima at 0.168 mol-EtOH (mol-*X*_EtOHox_)^−1^ h^−1^, 0.176 mol-CH_4_ (mol-*X*_Hym_)^−1^ h^−1^, and 0.066 mol-CH_4_ (mol-*X*_Acm_)^−1^ h^−1^ (Fig. 3a, b). During steady-state operation, no butanol formation was observed and therefore the biomass-specific butanol production rate (*q*_ButOH_) was zero. In course of the perturbation, however, *q*_ButOH_ increased to 0.015 ± 0.002 mol-ButOH (mol-*X*_EtOHox_)^−1^ h^−1^ on average and decreased only after the end of the perturbation experiment (Fig. 3c). After perturbation, *q*_EtOH_ and *q*_ButOH_ returned to values close to steady-state conditions while *q*_CH4,Hym_ and *q*_CH4,Acm_ remained low.

**Fig. 4:**
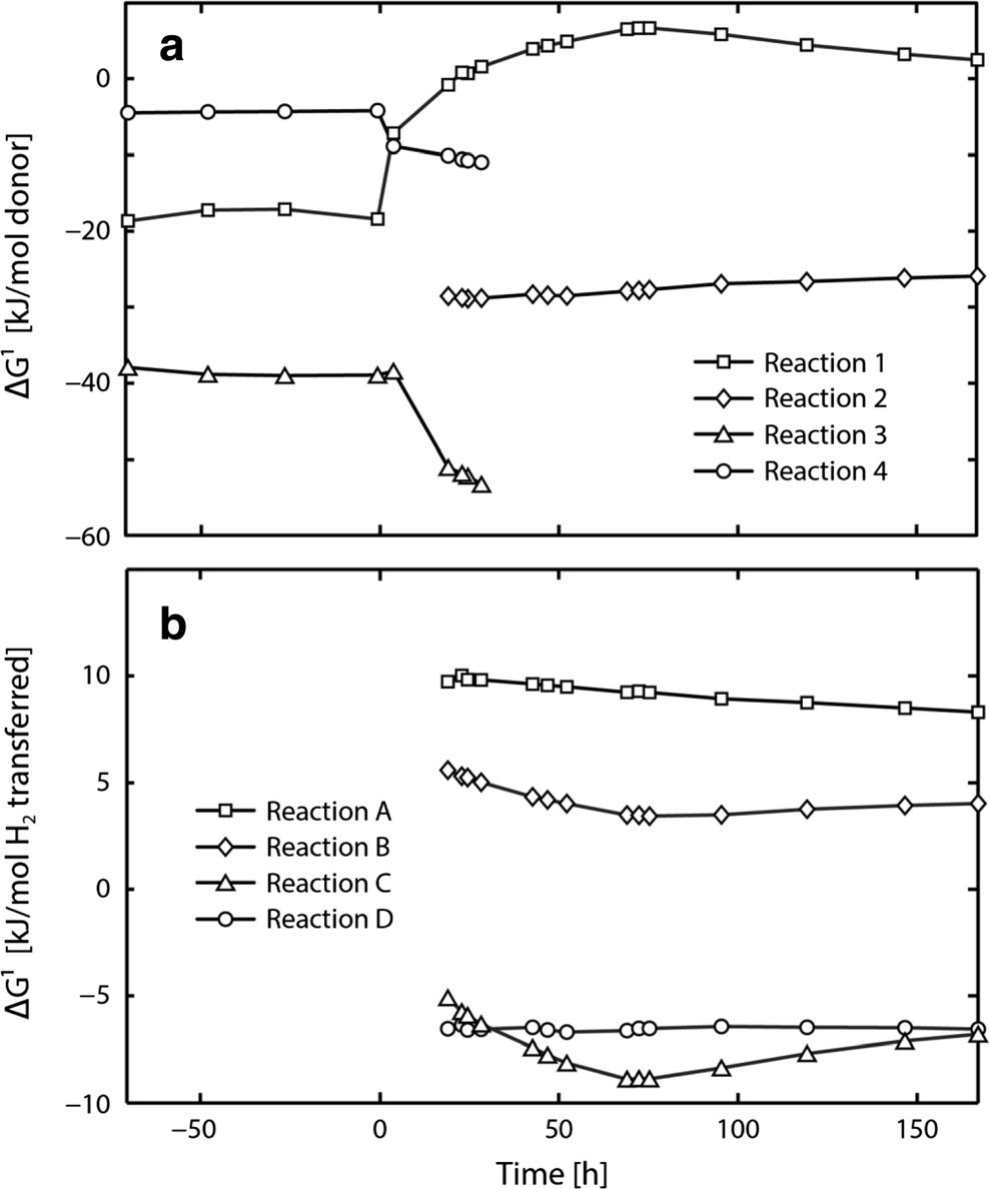
Actual Gibbs energy change of the partial reactions involved in syntrophic butyrate and ethanol conversion shown in Table 1 (**a**) and of possible butanol formation reactions shown in Table 2 (**b**) in course of experiment C1. *Reaction 1* butyrate conversion, *Reaction 2* ethanol conversion, *Reaction 3* acetoclastic methanogenesis, and *Reaction 4* hydrogenotrophic methanogenesis. For the Δ*G* ^1^ calculations, a temperature of 37 °C and a pH of 7.0 were used

Figure 4a shows the Δ*G*^1^ of the partial reactions involved in syntrophic butyrate and ethanol conversion in course of experiment C1. Prior to the perturbation experiment, all partial reactions shown in Table 1 were exergonic. Acetoclastic methanogenesis and anaerobic ethanol conversion were strongly thermodynamically favorable throughout the experiment. Anaerobic butyrate conversion became endergonic 20 h following perturbation. Energy sharing during syntrophic butyrate conversion was quantified as the Δ*G*^1^ (kJ mol^−1^-But) at steady-state conditions. Since the ethanol concentration was below the detection limit in this regime, the Δ*G*^1^ of ethanol conversion was non-quantifiable and therefore neglected in the calculation. The Δ*G*^1^ of the remaining reactions were normalized to 1 mol of butyrate using the stoichiometric yields. Unequal energy sharing between the butyrate-utilizing species (17 %), the hydrogenotrophic methanogens (9 %), and the acetoclastic methanogens (74 %) was found during steady-state operation of experiment C1.

To select the most likely butanol-forming reaction in the model, the Δ*G*^1^ of four possible catabolic reactions was investigated (Fig. 4b and Table 2). As shown in Fig. 4b, proposed reactions A and B were thermodynamically unfavorable while reactions C and D were exergonic throughout the experiment. Reactions C and D can be subdivided into two subsequent partial reactions. They share the same butanol formation reaction, namely reaction A, but differ by the initial partial reaction, which is either the reduction of acetate to butyrate via ethanol in case of reaction C or the conversion of ethanol to acetate and hydrogen in case of reaction D. Reaction D was chosen as the most likely butyrate formation reaction. This is because the enriched ethanol-utilizing species (see Online Resource Fig. S1) showed 93 % gene similarity to *P*. *acetylenicus* strain WoAcy1 which is known to convert the first partial reaction of reaction D (Schink 1985).

**Table 2:** Possible catabolic reactions involved in anaerobic butanol formation

No.	Reaction
A	H_2_ + 0.50 But + 0.50 H^+^ → 0.50 ButOH + 0.50 H_2_O
B	H_2_ + 0.50 Ac + 0.50 H^+^ → 0.25 ButOH + 0.75 H_2_O
C	H_2_ + 0.50 Ac + 0.50 H^+^ + 0.50 EtOH → 0.50 ButOH + H_2_O
D	0.50 EtOH + 0.50 But → 0.50 Ac + 0.50 ButOH

All reactions are defined per mole of hydrogen transferred. Reaction B is the combined reaction of acetate reduction to butyrate via hydrogen and reaction A. Reaction C is the combined reaction of acetate reduction to butyrate via ethanol and reaction A. Reaction D is the combined reaction of ethanol conversion to acetate and hydrogen and reaction A

### Experiment C2

**Fig. 5:**
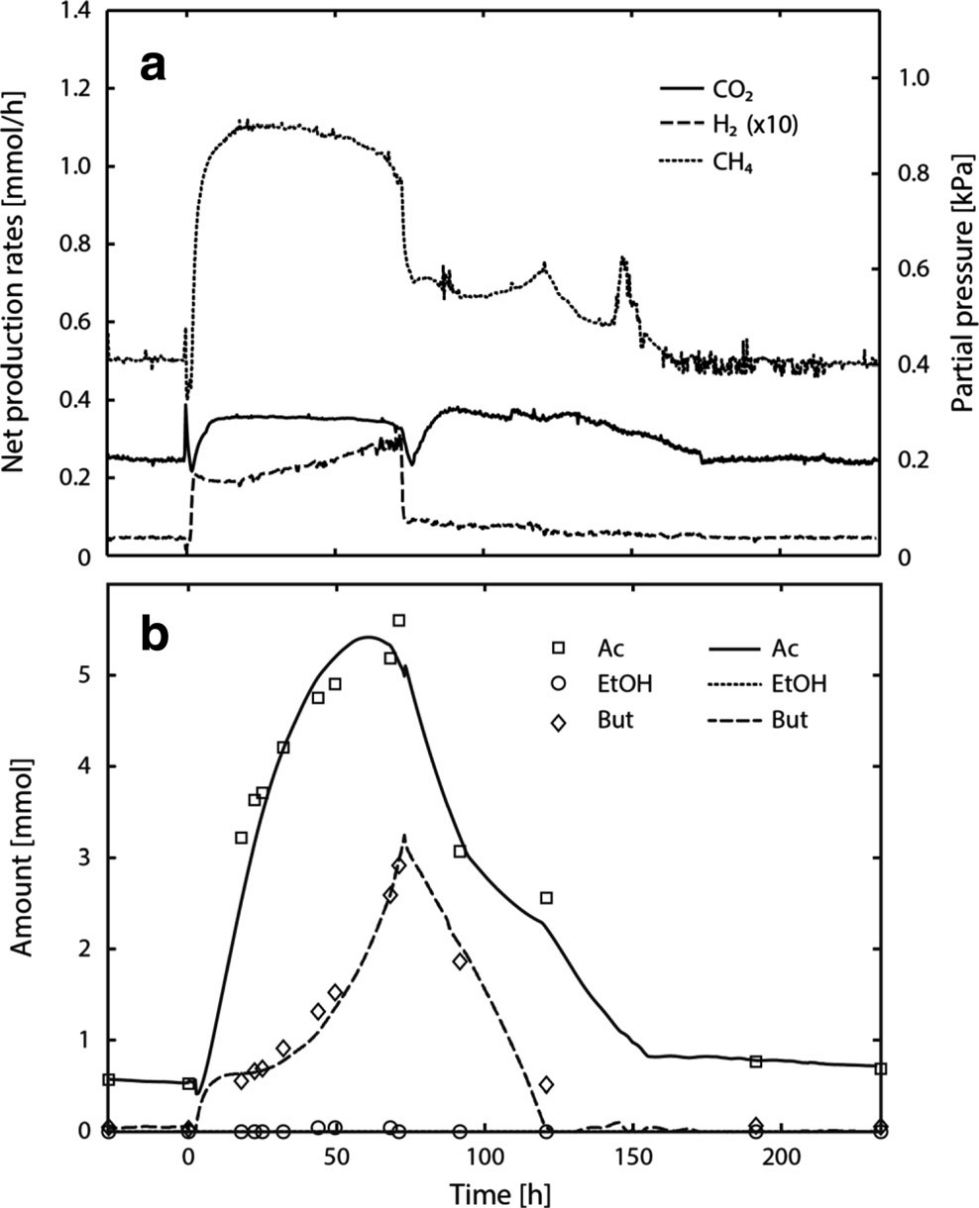
Measured gas production rates (**a**) and amounts in the reactor liquid (**b**) in course of experiment C2. Experiment C2 differed from experiment C1 by the dilution rate (0.0032 h^−1^) and the use of bicarbonate as base for pH control. In (**a**), the actual hydrogen partial pressure and hydrogen net production rate are obtained by dividing the displayed value by a factor of 10. In (**b**), measured amounts are indicated by *symbols* and model-derived amounts are represented by *lines*

To avoid bicarbonate-limiting conditions for hydrogenotrophic methanogenesis as previously observed in experiment C1, bicarbonate was supplied as base for pH control. Figure 5a shows the measured net production rates of methane, hydrogen, and carbon dioxide in course of experiment C2 and Fig. 5b shows the measured and model-derived amounts of butyrate, ethanol, and acetate. Model-based individual biomass amounts in course of experiment C2 are provided in the Online Resource Fig. S4. Steady-state conditions in experiment C2 were similar to experiment C1. Again, a CH_4_/CO_2_ ratio close to two was observed which is indicative of syntrophic butyrate and ethanol conversion. Shortly after perturbation (0 h), the methane and hydrogen partial pressure increased to 910 and 16 Pa, respectively. The hydrogen partial pressure increased slowly for 70 h and the methane partial pressure slowly decreased to approximately 810 Pa. The carbon dioxide partial pressure was 295 ± 3 Pa throughout the perturbation experiment. When the loading rate of the bioreactor was reduced to the original value, a strong decrease of the methane and hydrogen partial pressure was observed (70 h). At approximately 170 h, a CH_4_/CO_2_ ratio of two was reobtained, indicating the complete conversion of butyrate and ethanol to methane and carbon dioxide.

An accumulation of acetate (2.8 mM) and butyrate (1.5 mM) was observed following perturbation (Fig. 5b). However, compared to experiment C1, measured metabolite amounts were much lower. Only traces of ethanol (0.021 mM) and butanol (0.013 mM) were detected between 40 and 70 h of the perturbation experiment C2. The end of the perturbation experiment C2 was accompanied by a decrease of the acetate and butyrate concentration until steady-state concentrations were restored (170 h). This is in contrast to perturbation experiment C1, where acetate and butyrate continued to accumulate even after the end of perturbation.

**Fig. 6:**
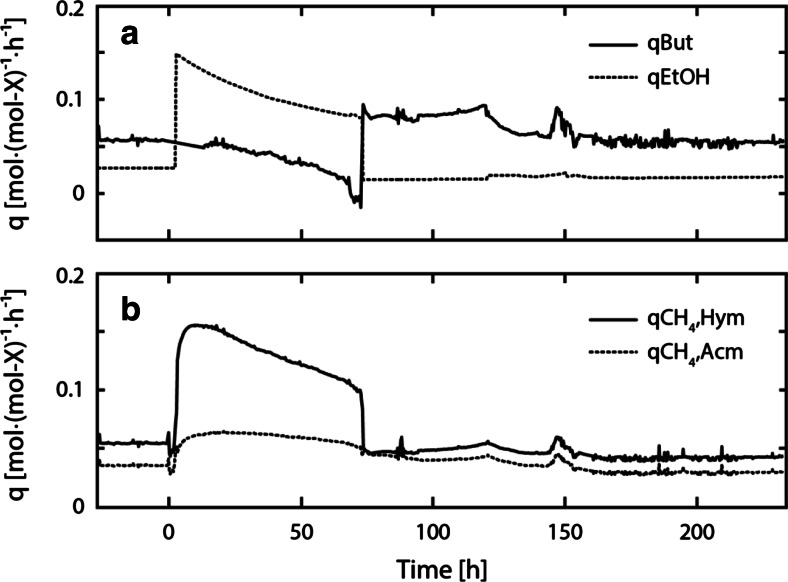
The biomass-specific butyrate consumption rate (*q*_But_) and biomass-specific ethanol consumption rate (*q*_EtOH_) in course of experiment C2 are shown in (**a**). The biomass-specific methane production rate of hydrogenotrophic methanogens (*q*_CH4,Hym_) and biomass-specific methane production rate of acetoclastic methanogens (*q*_CH4,Acm_) are shown in (**b**)

Figure 6 shows *q*_But_, *q*_EtOH_, *q*_CH4,Hym_, and *q*_CH4,Acm_ in course of experiment C2. Following perturbation, *q*_But_ decreased gradually until zero. Similar to observations made in experiment C1, *q*_EtOH_, *q*_CH4,Hym_, and *q*_CH4,Acm_ increased and peaked at 0.150 mol-EtOH (mol-*X*_EtOHox_)^−1^ h^−1^, 0.156 mol-CH_4_ (mol-*X*_Hym_)^−1^ h^−1^, and 0.064 mol-CH_4_ (mol-*X*_Acm_)^−1^ h^−1^. At the end of perturbation (70 h), *q*_But_ increased again and reached the initial steady-state value. Likewise, *q*_EtOH_, *q*_CH4,Hym_, and *q*_CH4,Acm_ returned to levels close to initial steady-state conditions.

**Fig. 7:**
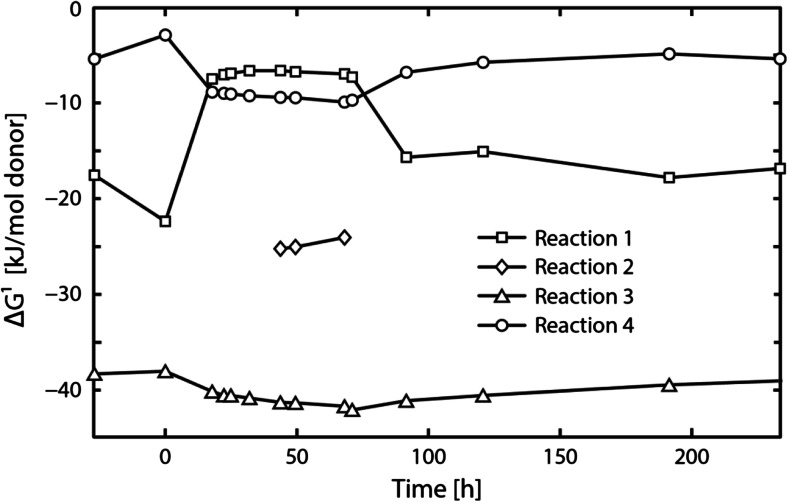
Actual Gibbs energy change of the partial reactions shown in Table 1, in course of experiment C2. *Reaction 1* butyrate conversion, *Reaction 2* ethanol conversion, *Reaction 3* acetoclastic methanogenesis, and *Reaction 4* hydrogenotrophic methanogenesis. For the Δ*G* ^1^ calculations, a temperature of 37 °C and a pH of 7.0 were used

Figure 7 shows the Δ*G*^1^ of the partial reactions involved in syntrophic butyrate and ethanol conversion in course of experiment C2. As opposed to experiment C1, all reactions shown in Table 1 were exergonic throughout experiment C2. The energy distribution between the butyrate-utilizing species (17 %), the hydrogenotrophic methanogens (10 %), and the acetoclastic methanogens (73 %) during steady-state operation was similar to experiment C1.

## Discussion

In this study, the combination of continuous cultivation, liquid measurements, online off-gas measurements, and model description successfully contributed to the identification of thermodynamic and kinetic control parameters during anaerobic butyrate conversion in ethanol and butyrate-fed methanogenic enrichments. An overview of the kinetic parameters determined and a comparison to literature is given in Online Resource Table S1. The growth yields estimated according to the Gibbs energy dissipation method were in the range of reported values. The *Y*_XC4ox/But_ of *S*. *cellicola* strain 19J-3, which showed 98 % gene similarity with the butyrate-utilizing species found in this study, has not yet been reported. However, a total biomass yield on butyrate has been reported for its closest relative, *Syntrophospora bryantii* DSM 3014T (94.6 % gene similarity), in coculture with different hydrogenotrophic methanogens (0.041–0.115 mol-*X*/mol-But, assuming 55 % protein content per gram dry weight) (Dong et al. 1994; Wu et al. 2006). The estimated total biomass yield of the enriched butyrate-utilizer and hydrogenotrophic methanogens on butyrate (0.088 mol-*X* mol-But^−1^) falls into the reported range.

To identify the microbial composition and to confirm the assumed catabolic reactions shown in Fig. 1, DGGE analysis was performed on samples taken prior to the perturbation experiments C1 and C2. As expected, a similar microbial composition was found in both experiments. All reactions shown in Fig. 1, except for acetoclastic methanogenesis, were identified based on the comparison of the gene similarities between the enriched species and the closest cultivated relative. Since a significant fraction of *Methanosaeta*-like species was observed using phase-contrast microscopy (Online Resource Fig. S2), acetoclastic methanogenesis was assumed as the acetate consuming reaction in the model. Syntrophic acetate-oxidizing bacteria can consume acetate in cooperation with hydrogenotrophic methanogens and are known to occur at conditions inhibitory to acetoclastic methanogens, e.g., high ammonium concentrations (>5.0 g L^−1^ NH_4_^+^-N) and high VFA levels (Schnürer et al. 1999; Westerholm et al. 2011). Such inhibitory conditions have not been observed in course of experiments C1 and C2. In addition, the retention time applied in this study (10–13 days) is rather short compared to the doubling times of acetate-oxidizing syntrophs, e.g., 20–25 days for *Clostridium ultunense* in coculture with a hydrogenotrophic methanogen under mesophilic conditions (Hattori 2008; Schnürer et al. 1997). These facts make a significant contribution of syntrophic acetate-oxidizers unlikely, although the reaction catalyzed by this microbial group, anaerobic acetate conversion, was exergonic throughout the experiments. The reverse pathway of anaerobic acetate oxidation, referred to as homoacetogenesis (reduction of CO_2_ by H_2_) was thermodynamically unfavorable throughout the two experiments. Therefore, syntrophic acetate conversion and homoacetogenesis have been neglected in the model description.

### Kinetic control of electron transfer

This study showed a clear influence of the hydrogen partial pressure on the biomass-specific flux of ethanol and butyrate conversion. A significant decrease of *q*_But_ was observed in perturbation experiment C2 even though anaerobic butyrate conversion remained exergonic. In the absence of significant product accumulation, the hydrogen partial pressure was the single parameter impacting reaction kinetics. The *q*_But_ clearly decreased as the hydrogen partial pressure increased during perturbation, and *q*_But_ increased again at the end of the perturbation when the initially low hydrogen partial pressure was restored. These conditions allowed to calculate a *K*_*i*,H2,C4ox_ of 0.074 ± 0.013 μM dissolved hydrogen (9 ± 2 Pa H_2_ in the gas phase) which is about ten times lower than the *K*_*i*,H2,C4ox_ proposed in the Anaerobic Digestion Model No. 1 (ADM1) (see Online Resource Table S1). ADM1 is a generalized anaerobic digestion model established by the IWA task group to provide a common platform for process description and further development (Batstone et al. 2002). The lower *K*_*i*,H2,C4ox_ obtained in this study supports that anaerobic butyrate conversion is already significantly inhibited at lower hydrogen partial pressures, as previously theoretically elaborated by Kleerebezem and Stams (2000).

Furthermore, an inhibitory effect of the hydrogen partial pressure on ethanol degradation was observed in both perturbation experiments. Eichler and Schink (1984) reported on hydrogen inhibition of anaerobic ethanol conversion in a pure culture of *Acetobacterium carbinolicum* strain WoProp1 grown on ethanol. Based on growth curves obtained either under H_2_/CO_2_ or N_2_/CO_2_ atmosphere (80 %/20 %), a *K*_*i*,H2,EtOH_ equal to 1.408 ± 0.253 μM of dissolved H_2_ was derived. The *K*_*i*,H2,EtOHox_ determined in this study (0.515 ± 0.022 μM dissolved H_2_ or 63 ± 3 Pa H_2_ in the gas phase, *R*^2^ = 0.983) was about three times lower, indicating a much stronger inhibitory effect of hydrogen on ethanol conversion. Moreover, in both experiments, the increasing hydrogen partial pressure was associated with decreasing *q*_CH4,Acm_ which suggests hydrogen inhibition on acetoclastic methanogenesis. However, based on the present experiments, it was not possible to either confirm or refute the effect of hydrogen on acetoclastic methanogenesis.

A tight coupling between hydrogen-producing and hydrogen-consuming organisms is essential to syntrophic methanogenic conversions. In this regard, the *K*_S,H2_ is an important kinetic parameter because a low *K*_S,H2_ permits efficient hydrogen uptake even at low hydrogen concentrations and reduces the inhibitory effect of hydrogen on the hydrogen-producing partner. The observed *q*_CH4,Hym_ of hydrogenotrophic methanogenesis was only one third of the *q*_CH4,Hym,max_ (0.500 mol-CH_4_ (mol-*X*_Hym_)^−1^ h^−1^) reported for *Methanobacterium flexile* strain GH and *M*. *subterraneum* strain A8p (Kotelnikova et al. 1998; Zhu et al. 2011), the two closest cultured relatives. These observations indicate that the hydrogenotrophic methanogens were operating below maximum capacity due to hydrogen limitation. Given above specific methane production rates, a *K*_S,H2_ of 52 ± 10 Pa (0.430 ± 0.082 μM dissolved H_2_) was deduced, which lies in the reported range for several hydrogenotrophic methanogens (see Online Resource Table S1). In line with previous findings in defined methanogenic cocultures on lactate and formate (Junicke et al. 2015a, b), an overcapacity of hydrogenotrophic methanogens was observed during syntrophic butyrate and ethanol conversion in non-defined methanogenic enrichments, reflecting the robustness of syntrophic bioconversions and enabling stable reactor performance.

In a chemostat the biomass-specific growth rate equals the dilution rate. Previous coculture studies on lactate showed that the syntrophic partners follow different strategies to adapt to a common biomass-specific growth rate (Junicke et al. 2015b). In the present study, the hydrogenotrophic methanogens compensated their low biomass yield per electron-mole of substrate (*Y*_*X*/e_) with a 2-fold higher biomass-specific electron transfer rate (*q*_e_), compared to the butyrate-utilizing partner. These findings provide further support for the previously reported growth strategies in defined methanogenic cocultures on lactate.

### Thermodynamic control of electron transfer

Thermodynamic analysis, combined with system modeling and reaction kinetics provides valuable insights into thermodynamic feasibility of the underlying reactions, pathway reversibility, and energy sharing between syntrophic partners.

In perturbation experiment C1, the increase of the hydrogen partial pressure was associated with an increasing actual Gibbs energy change of anaerobic butyrate conversion which became positive 20 h after increasing the influent ethanol concentration (Fig. 4a). At the same time *q*_But_ was effectively zero (Fig. 3a), providing experimental evidence for the thermodynamic control of anaerobic butyrate conversion and the need to implement thermodynamic restrictions in energy-limited anaerobic digestion models, as previously proposed in (Kleerebezem and Stams 2000; Kleerebezem and van Loosdrecht 2006). In anaerobic digestion models, such as ADM1, thermodynamic constraints are still neglected, thus violating thermodynamic principles.

By combining the model-derived *q* rates with thermodynamic analysis, it is furthermore possible to conclude on the reversibility of biochemical pathways. For example, a strongly negative *q*_But_ concomitant with a positive Δ*G*^1^ for butyrate conversion would reflect the reversibility of butyrate conversion. In experiment C1, however, *q*_But_ remained close to zero at positive Δ*G*^1^ for anaerobic butyrate conversion. These findings suggest that the reverse reaction of butyrate conversion did not occur. Pathway reversibility of anaerobic butyrate conversion was previously theoretically investigated by González-Cabaleiro et al. (2013). It was predicted that the reversibility of butyrate conversion is rather unlikely due to biochemical limitations, which agrees with the results of this study.

Since anaerobic bioconversions proceed close to thermodynamic equilibrium, it is of great interest to understand how thermodynamics affect the energy sharing among the syntrophic partners. Seitz et al. (1990b) investigated the energy distribution of defined methanogenic cocultures in ethanol-fed chemostats. They found an unequal distribution of the total Gibbs energy change between the hydrogen-producing acetogen (23 %) and the hydrogenotrophic methanogen (77 %). Unequal energy sharing was also demonstrated during syntrophic lactate conversion in different methanogenic cocultures (Junicke et al. 2015a, b). However, opposite to the results of Seitz et al. (1990b), the lactate-utilizing species shared a larger fraction of the total energy (79–83 %) compared to the hydrogenotrophic methanogen (17–21 %). This study revealed unequal energy distribution between the butyrate-utilizing species (17 %), the hydrogenotrophic methanogens (9–10 %), and the acetoclastic methanogens (73–74 %) during syntrophic butyrate conversion. As for the coculture study on lactate (Junicke et al. 2015a, b), a larger energy fraction was devoted to the hydrogen-producing acetogen while the hydrogenotrophic methanogen gained considerably less energy. The lower energy gain results in a low biomass yield which requires a larger *q*_e_ in order to maintain equal biomass-specific growth rates during syntrophic cooperation. Therefore, the different growth strategies are consistent with and directly follow from the unequal energy distribution between the syntrophic partners.

### Reduced product formation

Formation of reduced products occurs as a side-reaction in the presence of excess electrons. It provides an additional electron sink when (i) the enzymatic capacity of the primary reaction is exceeded, (ii) product inhibition occurs, (iii) the primary reaction becomes thermodynamically unfeasible, or (iv) the electron acceptor of the primary reaction becomes limiting. Since reduced products represent energetically dense chemicals, the conditions of their formation are focus of on-going research (González-Cabaleiro et al. 2013; Steinbusch et al. 2008; Steinbusch et al. 2011). So far, the role of electron transfer in the form of hydrogen remains unclear and it is unknown at which hydrogen partial pressure a switch between methanogenesis and reduced product formation occurs.

In experiment C1, butanol formation was observed 20 h following perturbation with increased ethanol concentrations, concomitant with increasing hydrogen partial pressures and decreasing carbon dioxide partial pressures (Fig. 2). The decrease of the carbon dioxide partial pressure led to bicarbonate limitation of hydrogenotrophic methanogenesis, and resulted in a further increase of the hydrogen partial pressure. The Δ*G*^1^ of butyrate conversion became positive at 20 h, accompanied by butanol production and ethanol accumulation. Ethanol conversion remained thermodynamically feasible during the perturbation experiment. Smith and McCarty (1989a, b) reported on similar observations in ethanol and propionate-perturbed CSTRs. They showed that the ethanol-oxidizing bacterium catalyzed the reduction of propionate with ethanol to propanol and acetate, and not the propionate-oxidizing bacterium. This was a striking observation since propionate conversion ceased due to elevated hydrogen partial pressures, and it was expected that the propionate-oxidizing bacterium would perform an alternative reaction to gain sufficient energy for growth. The formation of reduced products such as butanol reflects the redirection of electron fluxes towards an alternative electron acceptor when the hydrogenotrophic methanogen is limited. Smith and McCarty (1989b) argued that this mechanism may result in an altered overall stoichiometry, which is marked by lower hydrogen production in order to circumvent kinetic and thermodynamic limitations. Furthermore, they hypothesized that the increased ethanol consumption rate may increase the need for the use of alternative enzyme systems.

In the present study, butanol formation was observed at increasing hydrogen partial pressures after perturbation of the butyrate and ethanol-fed CSTR with increased ethanol concentrations. A shift from methanogenesis to reduced product formation was found when hydrogenotrophic methanogenesis was bicarbonate limited and when the hydrogen partial pressure exceeded 40 Pa. These findings imply that the hydrogen partial pressure may be an important control parameter to direct electron fluxes towards the formation of a valuable product such as butanol.

## Electronic Supplementary Material

ESM 1 (DOC 237 kb)
