# Supplementary material for: Kinetic and thermodynamic control of butyrate conversion in non-defined methanogenic communities
Source: Appl Microbiol Biotechnol. 2015 Sep 25;100:915–25. doi: 10.1007/s00253-015-6971-9 (PMC4703631; doi:10.1007/s00253-015-6971-9)
Supplement: Supplementary file 1 — (DOC 237 kb) [file 253_2015_6971_MOESM1_ESM.doc]

# SUPPLEMENTARY Material

**Title:** Kinetic and thermodynamic control of butyrate conversion in non-defined methanogenic communities

**Journal:** Applied Microbiology and Biotechnology

**Authors:** H. Junicke*, M.C.M. van Loosdrecht and R. Kleerebezem

**Affiliations:** ^1^ Department of Biotechnology, Delft University of Technology, Julianalaan 67, 2628 BC Delft, The Netherlands

* Corresponding author. Phone: +31 (0)15 27 81551. Email: h.junicke-2@tudelft.nl

# TABLES

**Table S1** Kinetic parameters determined in this study and compared to literature. *Y*_XC4ox/But_, biomass yield of the butyrate utilizer on butyrate; *Y*_XEtOHox/EtOH_, biomass yield of the ethanol utilizer on ethanol; *Y*_XAcm/CH4_, biomass yield of the acetoclastic methanogen on methane; *Y*_XHym/CH4_, biomass yield of the hydrogenotrophic methanogen on methane; *q*_But,max_, maximum biomass-specific butyrate consumption rate; *q*_EtOH,max_, maximum biomass-specific ethanol consumption rate; *q*_ButOH,max_, maximum biomass-specific butanol production rate; *K*_i,H2,C4ox_, 50 % inhibition constant of hydrogen on butyrate conversion; *K*_i,H2,EtOHox_, 50 % inhibition constant of hydrogen on ethanol conversion and *K*_S,H2_, affinity constant of hydrogenotrophic methanogens for hydrogen uptake.

| Parameter | Unit | Value | Ref. value | Ref. conditions | Reference |
| --- | --- | --- | --- | --- | --- |
| *Y*_XC4ox/But_ | mol-X_C4ox_/mol-But | 0.058 ± 0.009 | - | - | - |
| *Y*_XEtOHox/EtOH_ | mol-X_EtOHox_/mol-EtOH | 0.112 ± 0.017 | 0.090 | *Pelobacter acetylenicus* strain WoAcy1 in methanogenic coculture | Seitz et al. (1990b) |
| *Y*_XAcm/CH4_ | mol-X_Acm_/mol-CH_4_ | 0.092 ± 0.014 | 0.045 – 0.110 | acetoclastic methanogens | Huser et al. (1982); Hutten et al. (1980); Smith and Mah (1978); Weimer and Zeikus (1978) |
| *Y*_XHym/CH4_ | mol-X_Hym_/mol-CH_4_ | 0.058 ± 0.009 | 0.056 – 0.136 | *Methanobacterium* species | Fardeau and Belaich (1986); Leadbetter and Breznak (1996); Morii et al. (1987); Roberton and Wolfe (1970); Robinson and Tiedje (1984); Schönheit et al. (1980); Seitz et al. (1990b) |
| *q*_EtOH,max_ | mol‑EtOH∙ (mol‑X_EtOHox_)^‑1^∙h^-1^ | 0.222 ± 0.032 | 0.232 – 1.389 | ethanol-utilizing species | Müller (2010); Seitz et al. (1990a) |
| *q*_ButOH,max_ | mol-ButOH∙ (mol-X_EtOHox_)^‑1^∙h^-1^ | 0.015 ± 0.002 | - | - | - |
| *K*_i,H2,C4ox_ | µM dissolved H_2_ | 0.074 ± 0.013 | 0.706 |  | Batstone et al. (2002) |
| *K*_i,H2,EtOHox_ | µM dissolved H_2_ | 0.515 ± 0.022 | 1.408 ± 0.253 | *Acetobacterium carbinolicum* strain WoProp1 | Eichler and Schink (1984) |
| *K*_S,H2_ | µM dissolved H_2_ | 0.430 ± 0.082 | 0.4 – 6.0 |  | Goodwin et al. (1991); Junicke et al. (2015a); Kristjansson et al. (1982); Robinson and Tiedje (1984); Schauer et al. (1982) |

# FIGURES


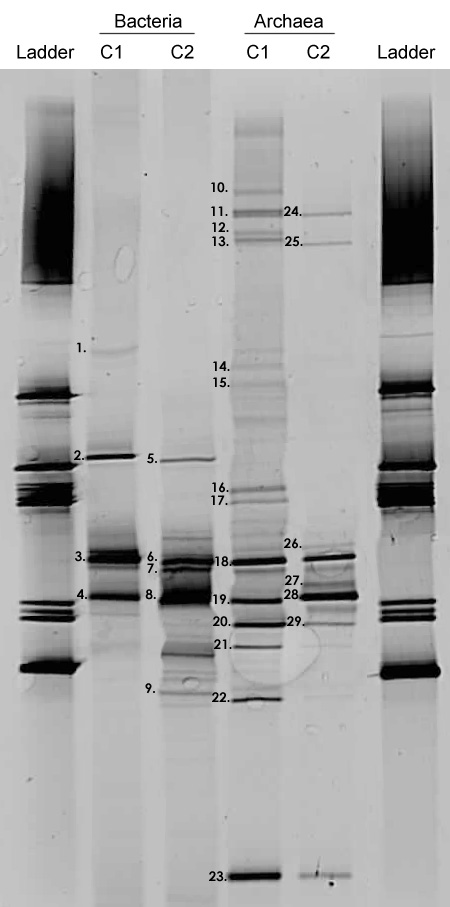


**Fig. S1** DGGE results of bacterial and archaeal 16S rDNA gene. Samples were taken prior to the perturbation experiments C1 and C2. Band numbers 1 – 29 represent successfully sequenced fragments and correspond to GenBank accession numbers KR349066 – KR349094


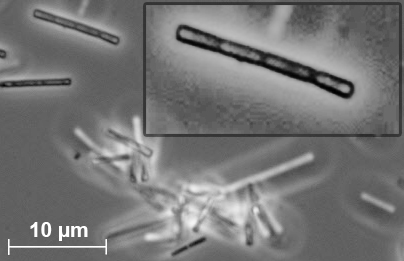


**Fig. S2** Phase-contrast micrograph of the butyrate and ethanol-fed enrichment during steady-state operation. The enlarged inset resembles a typical *Methanosaeta*-like cell, operating as the acetoclastic methanogen in the present study

**
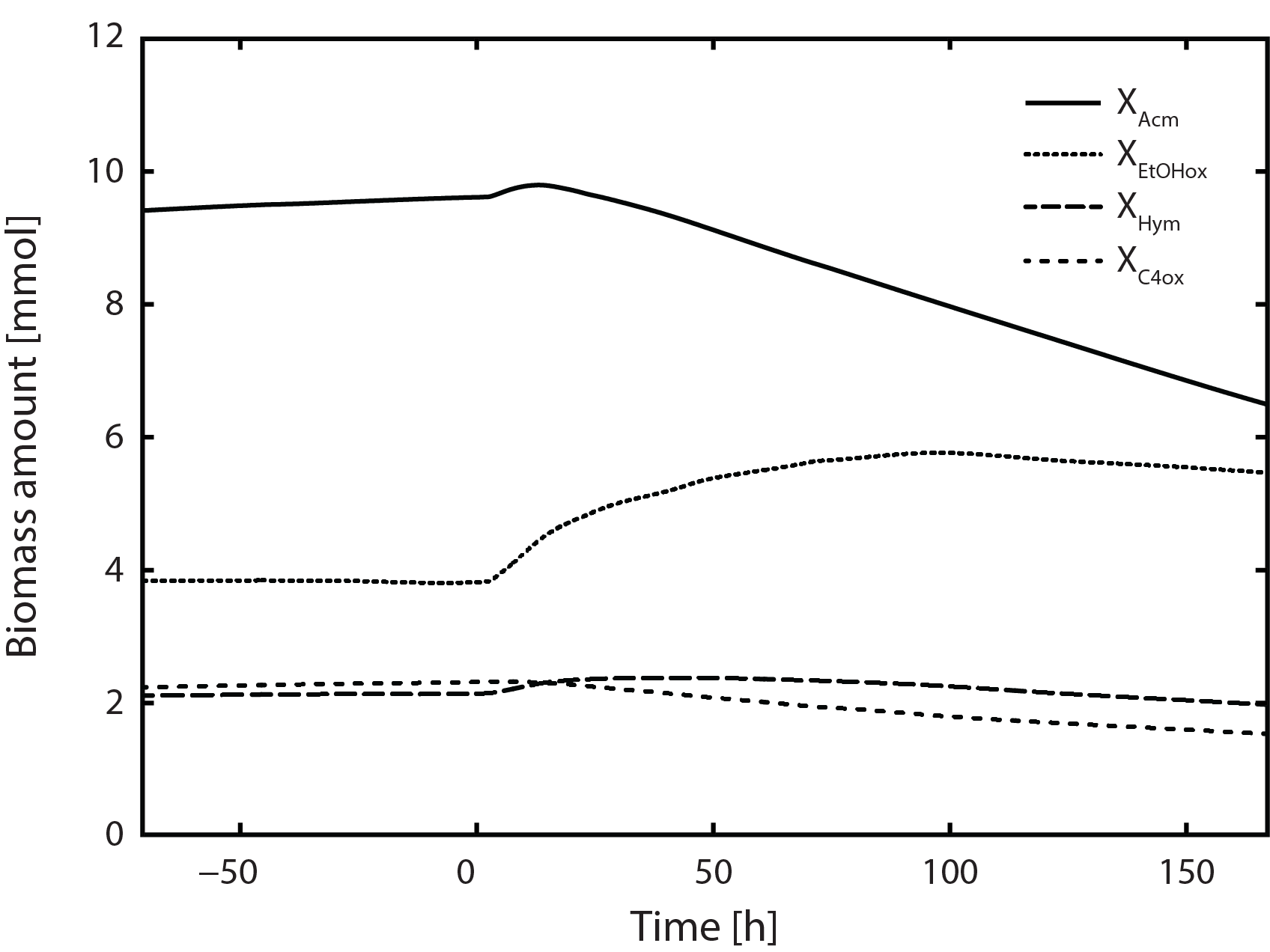
**

**Fig. S3** Model-derived individual biomass amounts in course of experiment C1 shown in Fig. 2. X_Acm_, biomass of acetoclastic methanogens catalysing reaction 3 in Table 1; X_EtOHox_, biomass of ethanol-utilizing species catalysing reaction 2 in Table 1; X_Hym_, biomass of hydrogenotrophic methanogens catalysing reaction 4 in Table 1; X_C4ox_, biomass of butyrate-utilizing species catalysing reaction 1 in Table 1


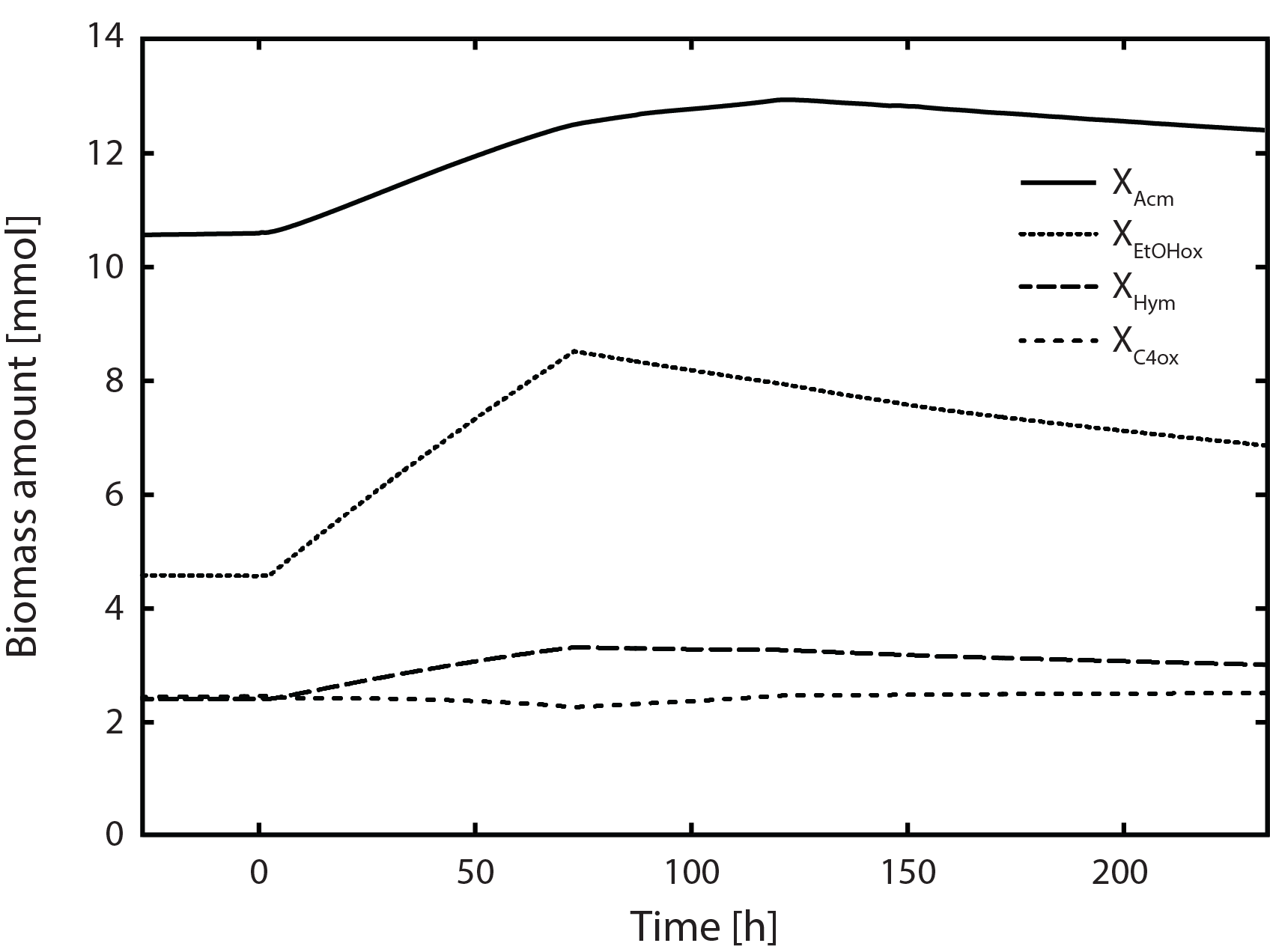


**Fig. S4** Model-derived individual biomass amounts in course of experiment C2 shown in Fig. 5. X_Acm_, biomass of acetoclastic methanogens catalysing reaction 3 in Table 1; X_EtOHox_, biomass of ethanol-utilizing species catalysing reaction 2 in Table 1; X_Hym_, biomass of hydrogenotrophic methanogens catalysing reaction 4 in Table 1; X_C4ox_, biomass of butyrate-utilizing species catalysing reaction 1 in Table 1
